# Presence of heat shock protein 47-positive fibroblasts in cancer stroma is associated with increased risk of postoperative recurrence in patients with lung cancer

**DOI:** 10.1186/s12931-020-01490-1

**Published:** 2020-09-14

**Authors:** Takuto Miyamura, Noriho Sakamoto, Kaori Ishida, Tomoyuki Kakugawa, Hirokazu Taniguchi, Yoshiko Akiyama, Daisuke Okuno, Atsuko Hara, Takashi Kido, Hiroshi Ishimoto, Takuro Miyazaki, Keitaro Matsumoto, Tomoshi Tsuchiya, Hiroyuki Yamaguchi, Taiga Miyazaki, Yasushi Obase, Yuji Ishimatsu, Takeshi Nagayasu, Hiroshi Mukae

**Affiliations:** 1 Department of Respiratory Medicine, Nagasaki University Graduate School of Biomedical Sciences, 1-7-1 Sakamoto, Nagasaki, 852-8501 Japan; 2 Department of Pathology and Laboratory Medicine, Kansai Medical University, 2-5-1 Shinmachi, Hirakata City, Osaka 574-1191 Japan; 3 Department of Pulmonology and Gerontology, Graduate School of Medicine, Yamaguchi University, 1-1-1 Minamikogushi, Ube City, Yamaguchi 755-8505 Japan; 4 Molecular Pharmacology Program and Department of Medicine, Memorial Sloan Kettering Cancer Center, 1275 York Avenue, New York, NY10065 USA; 5 Department of Surgical Oncology, Nagasaki University Graduate School of Biomedical Sciences, 1-7-1 Sakamoto, Nagasaki, 852-8501 Japan; 6 Department of Nursing, Nagasaki University Graduate School of Biomedical Sciences, 1-7-1 Sakamoto, Nagasaki, 852-8520 Japan

**Keywords:** Lung cancer, Molecular biology, Pathology, Pulmonary fibrosis, Thoracic surgery

## Abstract

**Background:**

Heat shock protein 47 (HSP47), a collagen-binding protein, has a specific role in the intracellular processing of procollagen production. HSP47 expression is associated with cancer growth and metastasis in several types of cancers. However, none of the studies have assessed whether HSP47 expression is associated with the risk of postoperative recurrence of lung cancer until now. Therefore, we aimed to assess this association.

**Methods:**

The study population consisted of a cohort of consecutive patients who underwent surgery for lung cancer at Nagasaki University Hospital, Nagasaki, Japan, from January 2009 to December 2010. Patient characteristics, survival and disease-free survival (DFS), and laboratory findings were compared between patients who tested positive and negative for HSP47 expression in lung cancer cells and between those who showed high and low numbers of HSP47-positive fibroblasts in cancer stroma.

**Results:**

A total of 133 patients underwent surgery for lung cancer. Sixty-seven patients (50.4%) had HSP47-positive cancer cells, and 91 patients (68.4%) had a higher number of HSP47-positive fibroblasts. The patients with a high number of HSP47-positive fibroblasts had a shorter DFS than those with a low number of HSP47-positive fibroblasts. Multivariate analysis identified only the presence of a high number of HSP47-positive fibroblasts as an independent risk factor for recurrence of lung cancer after surgery (odds ratio, 4.371; 95% confidence interval, 1.054–29.83; *P* = 0.042).

**Conclusion:**

The present study demonstrated that the presence of a high number of HSP47-positive fibroblasts in the cancer stroma was a risk factor for recurrence of lung cancer after surgery.

## Background

Heat shock protein 47 (HSP47) is a collagen-binding, stress-inducible protein localized in the endoplasmic reticulum, and it plays a specific role in the intracellular processing of procollagen production as a collagen-specific molecular chaperone [1]. HSP47 expression is reported to be correlated with collagen expression. Therefore, HSP47 is strongly associated with collagen-related fibrotic diseases, including liver, heart, kidney, and pulmonary fibrosis [2]. We have reported that HSP47 expression is upregulated and associated with the progression of pulmonary fibrosis and pulmonary fibrotic disorders in animal models and human patients [3–7].

HSP47 has also been reported to be associated with several types of cancers, including cervical, breast, pancreatic, gastric, and colon cancer [8–11]. It is encoded by the *SERPINH1* gene located on chromosome 11q13.5; this region is one of the most frequently amplified in human cancer [12]. Several types of cancers are associated with abnormal protein folding, and HSP47 has been described as an important chaperone in the control and maintenance of cellular protein homeostasis [13]. Furthermore, HSP47 expression promotes cancer progression in part by enhancing the deposition of extracellular matrix (ECM) proteins, including collagens.

In lung cancers, expression of HSP47 in squamous cell carcinomas is higher than that in normal human bronchial epithelium cells [14]. However, no previous study has discussed the clinical significance of HSP47 expression in lung cancer. Therefore, this study aimed to reveal the association between the expression of HSP47 and lung cancer.

## Methods

### Study population and covariates

The study protocol was approved by the Institutional Review Board at Nagasaki University Hospital (approval number 17112009) and conducted in accordance with the Declaration of Helsinki. Informed consent was not required in view of the retrospective study design and the anonymity of the patient records reviewed, pursuant to the ethical guidelines of the Japanese Ministry of Health, Labor, and Welfare. The study population consisted of a cohort of consecutive patients who underwent surgery for lung cancer at Nagasaki University Hospital, Nagasaki, Japan, from January 2009 to December 2010. Patient characteristics, overall survival (OS), disease-free survival (DFS), and laboratory findings obtained from the clinical records were compared between patients showing pathologically positive and negative HSP47 expression in lung cancer cells, and high and low numbers of fibroblasts in the cancer stroma. The observation period was defined from surgery to December 2018.

### Immunohistochemistry

Formalin-fixed, paraffin-embedded sections (thickness, 3 μm) were prepared from surgical specimens of patients with lung cancer. After deparaffinization and dehydration, the sections were placed in pH 9.0 ethylenediaminetetraacetic acid buffer and autoclaved at 95 °C for 30 min for epitope retrieval. The sections were incubated in 3% hydrogen peroxide for 10 min to block endogenous peroxidase activity. After incubation in blocking solutions, the sections were washed twice in tris-buffered saline (TBS) and incubated with anti-HSP47 antibodies (diluted 1:15000; M16.10A1, ADI-SPA-470; Enzo life sciences) at room temperature for 60 min. Sections were washed in TBS two times and incubated with peroxidase-labeled anti-rabbit or anti-mouse antibodies (Histofine Simplestain Max PO; Nichirei) for 30 min at room temperature. Peroxidase activity was detected with diaminobenzidine (Sigma-Aldrich). Sections were counterstained with hematoxylin and dehydrated.

### HSP47 expression scores in lung cancer cells

**Fig. 1:**
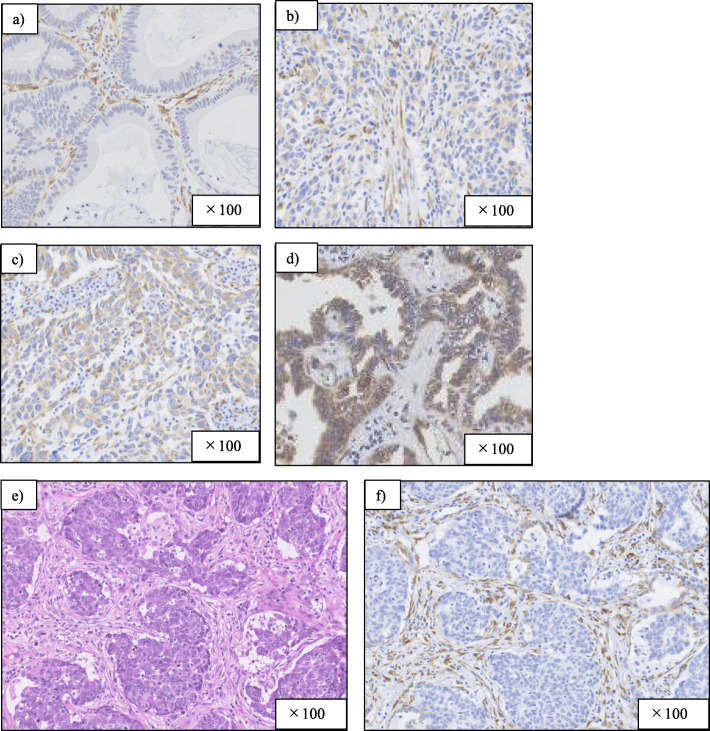
Extent of HSP47 immunostaining in lung cancer (**a**) score 0: no immunostaining; **b** 1: weak staining; **c** 2: moderate staining; and **d** 3: strong staining. Fibroblasts in cancer stroma (**e**) Hematoxylin-Eosin staining, (**f**) HSP47 immunostaining

Each slide was observed by scanning the entire tissue specimen. One pathologist (KI) and one respiratory physician (TM) without prior knowledge of the patient’s clinical characteristics evaluated the HSP47 immunoreactivity of lung cancer cells. Staining intensity was scored as follows: no staining, 0; weak staining, 1; moderate staining, 2; and strong staining, 3 (Fig. 1 a–d). The extent of HSP47 staining was scored according to the percentage of HSP47-expressing lung cancer cells. HSP47 expression in lung cancer cells was categorized as positive or negative based on the intensity and extent scores (Table 1). The evaluators used a distal microscope (Nano Zoomer Distal Pathology. view 2).

**Table 1:** Expressions of HSP47 in lung cancer cells

Expression	Conditions
Positive	> = 50% of all cancer cells express score 2 or 3
Negative	1–50% of all cancer cells express score 2 or 3
	All cancer cells express score 0 or 1

### Quantitation of HSP47-positive fibroblasts in cancer stroma

In cancer stroma, the number of fibroblasts that reacted with the anti-HSP47 antibodies were counted using a distal microscope (Nano Zoomer Distal Pathology. view 2), and images of cells in three 1-mm^2^ fields were acquired at 400× magnification. The number of fibroblasts was represented as the mean value of the three fields, according to the methodology of a previous study [11].

### Lung cancer staging

Lung cancer stage was based on the IASLC lung cancer staging project: Proposals for revision of the TNM stage groupings in the forthcoming (eighth) edition of TNM classification for lung cancer [15]. All TNM classifications and stages were determined based on pathological findings.

### Statistical analysis

Categorical variables are presented as frequency and quantitative variables are presented as the median and interquartile range (IQR). Univariate tests (Wilcoxon rank-sum and chi-squared tests) and univariate and multivariate logistic regression analyses were performed to identify differences between patients who showed positive and negative HSP47 expression in lung cancer, high and low numbers of HSP47-positive fibroblasts in cancer stroma, and possible risk factors for primary disease death and recurrence. Receiver operating characteristic (ROC) curves were generated to determine cut-off values of risk factors for recurrence. The cut-off values were determined by the maximum value of sensitivity - (1 - specificity) for recurrence or death by the value of HSP47-positive fibroblasts. The Kaplan–Meier method was used to determine the cumulative probability of OS and DFS, and the differences were evaluated using log-rank tests. Firth penalized logistic regression was used in the analyses of possible risk factors for primary disease-related death of male patients and of those with M classification (M1) because of quasi-complete separation. All *P*-values were two-sided and considered statistically significant when they were less than 0.05. All the statistical analyses were performed using the JMP Pro software program (version 14.0.0; SAS Institute, Inc., Cary, NC, USA).

## Results

### Participant characteristics

The study population consisted of 133 patients who underwent surgery for lung cancer. The characteristics of the patients are shown in Table 2. The median patient age was 70 years (male patients, 87 (65.4%)). Seventy-six patients had a history of smoking (61.8%), and 99 patients showed adenocarcinoma (74.4%). The percentages of patients showing each pathological stage of lung cancer were as follows: stage I, 77.4%; stage II, 14.2%; stage III, 7.6%; and stage IV, 0.9%.

**Table 2:** Characteristics of patients who underwent surgery for lung cancer

Characteristics		% or (IQR)	[n]
N, no	133		
Age, median	70	(62.0–77.0)	[133]
Sex Male, no	87	65.4%	[133]
BMI (kg/m^2^), median	22.8	(20.9–24.7)	[133]
History of smoking, no	76	61.8%	[123]
Brinkman index, median	900	(500–1393)	[81]
Pulmonary function test			
%VC, median (%)	110.7	(100.9–120.6)	[103]
FEV1/FVC, median (%)	73.9	(64.7–77.6)	[103]
%DLCO, median (%)	88.6	(75.7–105.7)	[98]
KL-6, median (U/mL)	234	(169.5–321)	[81]
Orgnization type of lung cancer			
Adenocarcinoma, no	99	74.4%	[133]
Squamous cell carcinoma, no	24	18.0%	[133]
Small cell caricnoma, no	4	3.0%	[133]
LCNEC, no	4	3.0%	[133]
Adenosquamous cell carcinoma, no	2	1.5%	[133]
Tumor size (mm)	23	(15–32.5)	[133]
pT classification, no (T3, T4)	28	26.4%	[133]
pN classification, no (N1, N2)	23	17.3%	[133]
pM classification, no (M1)	2	1.5%	[133]
pStage			
I	100	77.4%	[133]
II	19	14.2%	[133]
III	12	7.6%	[133]
IV	2	0.9%	[133]

*IQR* interquartile range, *BMI* body mass index, *VC* vital capacity, *FEV1/FVC* forced expiratory volume in one second/forced vital capacity, *DLCO* diffusing capacity for carbon monoxide, *KL-6* Klebs von den Lungen-6, *LCNEC* Large cell neuroendocrine carcinoma

### Comparison of patient characteristics between patients with HSP47-positive and HSP47-negative lung cancer cells

Sixty-seven patients (50.4%) had HSP47-positive cancer cells (Table 3). Patients with HSP47-positive cancer cells showed a greater frequency of smoking history (48.4% vs. 27.9%), higher percentage of diffusing capacity for carbon monoxide (97.6% vs. 80.9%), and a greater frequency of adenocarcinomas (89.6% vs. 59.1%) than those with HSP47-negative cancer cells. In contrast, serum KL-6 levels (193 U/mL vs. 263 U/mL) and the number of HSP47-positive fibroblasts in the cancer stroma (71 vs. 125) were lower in the HSP47-positive group than in the HSP47-negative group.

**Table 3:** Characteristics of patients divided by HSP47 expression in cancer cells or number of HSP47-positive fibroblasts in patients with lung cancer

Characteristics	HSP47 expresion in cancer cells						*P* value	Number of HSP47-positive fibroblasts						*P* value
	Positive			Negative				High			Low			
		% or (IQR)	[n]		% or (IQR)	[n]			% or (IQR)	[n]		% or (IQR)	[n]	
N, no	67	50.4%		66	49.6%			91	68.4%		42	31.6%		
Age, median	69	(61.0–77.0)	[67]	74	(63.0–77.0)	[66]	*p* = 0.341	71	(63.0–77.0)	[91]	68.5	(61.0–77.0)	[42]	*p* = 0.673
Sex Male, no	41	61.2%	[67]	46	69.7%	[66]	*p* = 0.363	68	74.7%	[91]	19	45.2%	[42]	*p* = 0.015*
BMI (kg/m^2^), median	22.8	(20.9–25.0)	[67]	22.8	(20.8–24.5)	[66]	*p* = 0.968	22.9	(21.5–25.0)	[91]	21.9	(20.7–24.0)	[42]	*p* = 0.263
History of smoking, no	30	48.4%	[62]	17	27.9%	[61]	*p* = 0.026*	61	71.8%	[85]	15	39.5%	[38]	*p* = 0.012*
Brinkman index, median	877.5	(455–1515)	[30]	900	(562–1200)	[17]	*p* = 0.322	900	(680–1500)	[61]	620	(275–1125)	[15]	*p* = 0.042*
Pulmonary function test														
%VC, median (%)	111.4	(102.3–120.4)	[53]	109	(98.2–122.3)	[50]	*p* = 0.611	106.4	(94.0–120.1)	[68]	115	(104.1–123.2)	[35]	*p* = 0.041*
FEV1/FVC, median (%)	73.9	(64.4–80.3)	[53]	73.4	(66.8–77.0)	[50]	*p* = 0.559	73.5	(64.8–79.0)	[68]	74.6	(64.5–77.4)	[35]	*p* = 0.983
%DLCO, median (%)	97.6	(82.1–109.9)	[50]	80.9	(65.0–97.1)	[48]	*p* = 0.001*	84.3	(73.4–104.3)	[65]	93.4	(77.0–109.1)	[33]	*p* = 0.371
KL-6, median (U/mL)	193	(152.0–303.5)	[37]	263	(178–370.3)	[44]	*p* = 0.020*	247	(171–345.5)	[56]	231	(167.5–316.5)	[16]	*p* = 0.377
Adenocarcinoma, no	60	89.6%	[67]	39	59.1%	[66]	*p <* 0.001*	57	62.6%	[91]	42	100.0%	[42]	*p <* 0.001*
pT classification, no (T3, T4)	2	3.0%	[67]	6	9.1%	[66]	*p* = 0.165	8	8.8%	[91]	0	0.0%	[42]	*p* = 0.557
pN classification, no (N1, N2)	8	11.9%	[67]	15	22.7%	[66]	*p* = 0.114	21	23.1%	[91]	2	4.8%	[42]	*p* = 0.012*
pM classification, no (M1)	1	1.5%	[67]	1	1.5%	[66]	*p* = 1.000	2	2.2%	[91]	0	0.0%	[42]	*p* = 1.000
pStage							*p* = 0.125							*p* = 0.025*
I	56	83.6%	[67]	44	66.7%	[66]		62	68.1%	[91]	38	90.5%	[42]	
II	7	10.5%	[67]	12	18.2%	[66]		15	16.5%	[91]	4	9.5%	[42]	
III	3	4.5%	[67]	9	13.7%	[66]		12	13.2%	[91]	0	0.0%	[42]	
IV	1	1.5%	[67]	1	1.5%	[66]		2	2.2%	[91]	0	0.0%	[42]	
Recurrence, no	13	19.4%	[67]	16	24.2%	[66]	*p* = 0.535	27	29.7%	[91]	2	4.8%	[42]	*p* = 0.001*
Primary disease death, no	12	17.9%	[67]	7	10.6%	[66]	*p* = 0.3221	15	16.5%	[91]	3	7.1%	[42]	*p* = 0.173
HSP47 expression positive in cancer cells, no	–	–		–	–			39	42.9%	[91]	28	66.7%	[42]	*p =* 0.015*
HSP47-positive fibroblasts, no	71.0	(28.5–117.5)	[67]	125	(74.1–180.6)	[66]	*p <* 0.001*	–	–		–	–		

^*^; *P* value < 0.05, Fisher’s exact test or Wilcoxon test

*IQR* interquartile range, *BMI* body mass index, *VC* vital capacity, *FEV1/FVC* forced expiratory volume in one second/forced vital capacity, *DLCO* diffusing capacity for carbon monoxide, *KL-6* Klebs von den Lungen-6

### Comparison of patient characteristics between patients with high and low number of HSP47-positive fibroblasts in the cancer stroma

The optimum cut-off level for discriminating between high and low numbers of HSP47-positive fibroblasts was 73. Thus, 91 patients (68.4%) had a high number of HSP47-positive fibroblasts (Table 3). The group with a high number of HSP47-positive fibroblasts had a greater proportion of male patients (74.7% vs 45.2%), patients with a history of smoking (71.8% vs 39.5%), patients with pN1 or pN2 classification (23.1% vs 4.8%), and patients who showed recurrence (29.7% vs 4.8%) and higher scores on the Brinkman index (900 vs. 620) than those in the group with a low number of HSP47-positive fibroblasts. However, the proportion of patients who had adenocarcinoma was lower in the group with a high number of HSP47-positive fibroblasts (62.6% vs 100.0%) than in the group with a low number of HSP47-positive fibroblasts.

### Survival curve and risk factors for recurrence and death

**Fig. 2:**
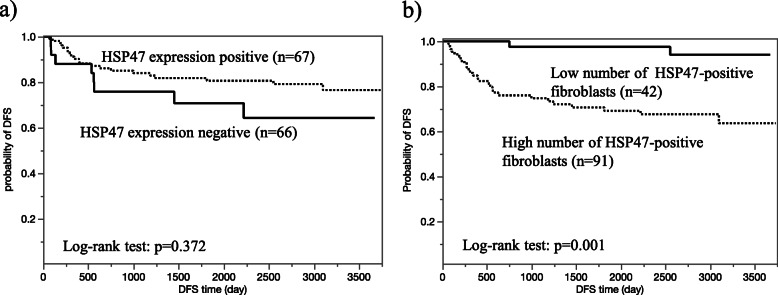
Survival curves of patients with lung cancer according to HSP47 expression in lung cancer cells or the number of HSP47-positive fibroblasts in the stroma. **a** There was no significant difference in disease-free survival (DFS) curves of patients showing HSP47-positive or HSP47-negative cancer cells (*p* = 0.372, log-rank test). **b** Patients with a high number of HSP47-positive fibroblasts had significantly shorter DFS (*p* = 0.001, log-rank test, cut-off value = 73)

Figure 2 shows the DFS curves according to the presence or absence of HSP47 expression in cancer cells, and high or low numbers of HSP47-positive fibroblasts in the cancer stroma. The HSP47-positive and HSP47-negative groups showed no significant differences (*p* = 0.372, log-rank test). However, patients with a high number of HSP47-positive fibroblasts had a shorter DFS than those with few HSP47-positive fibroblasts (*p* = 0.001, log-rank test). Logistic regression analysis identified male sex (odds ratio [OR] 6.109, 95% confidence interval [CI] 1.987–26.75, *p* < 0.001), history of smoking (OR 4.665 95% CI 1.642–16.82, *p* = 0.003), and a high number of HSP47-positive fibroblasts in the cancer stroma (OR 8.437, 95% CI 2.351–54.07, *p* = 0.004) as significant risk factors for the recurrence of lung cancer after surgery (Table 4). In contrast, logistic regression analysis revealed adenocarcinoma to be inversely associated with recurrence (OR 0.362, 95% CI 0.149–0.886, *p* = 0.027). However, multivariate analysis identified only a high number of HSP47-positive fibroblasts in the cancer stroma as an independent risk factor for recurrence of lung cancer after surgery (OR 4.371, 95% CI 1.054–29.83, *p* = 0.042) (Table 4).

**Table 4:** Risk factors for recurrence of lung cancer

Characteristics	Univariate analysis			Multivariate analysis		
	OR	(95% CI)	*P* value	OR	(95% CI)	*P* value
Age	1.015	(0.9717–1.063)	*p* = 0.498			
Sex Male	6.109	(1.987–26.75)	*p <* 0.001*	3.071	(0.611–18.84)	*p* = 0.176
BMI (kg/m^2^)	1.082	(0.919–1.304)	*p* = 0.351			
History of smoking	4.665	(1.642–16.82)	*p =* 0.003*	1.478	(0.321–7.852)	*p* = 0.622
Pulmonary function test						
%VC	0.986	(0.960–1.000)	*p* = 0.121			
FEV1/FVC	0.958	(0.934–1.019)	*p* = 0.264			
%DLCO	0.991	(0.971–1.000)	*p* = 0.244			
KL-6	1.002	(0.999–1.006)	*p* = 0.175			
Adenocarcinoma	0.362	(0.149–0.886)	*p =* 0.027*	0.733	(0.263–2.037)	*p* = 0.550
HSP47 expression in lung cancer (Positive)	0.586	(0.230–1.586)	*p* = 0.283			
Number of HSP47-positive fibroblasts (High)	8.437	(2.351–54.07)	*p =* 0.004*	4.371	(1.054–29.83)	*p =* 0.042*

*; *P* value < 0.05, Logistic regression analysis

*OR* odds ratio, *CI* confidence interval, *BMI* body mass index, *DLCO* diffusing capacity for carbon monoxide

The OS curves based on HSP47 expression in cancer cells and high or low numbers of HSP47-positive fibroblasts are shown in Additional file 1. There was no significant difference between the HSP47-positive and HSP47-negative groups (*p* = 0.388, log-rank test). However, patients with a high number of HSP47-positive fibroblasts had a shorter OS than those with a low number of HSP47-positive fibroblasts (*p* < 0.001, log-rank test, cut-off value = 123). Logistic regression analysis identified age (OR 1.055, 95% CI 1.007–1.109, *p* = 0.028), male sex (OR 22.50, 95% CI 4.535–408.2, *p <* 0.001), history of smoking (OR 11.70, 95% CI 3.243–75.17, *p <* 0.001), and a high number of HSP47-positive fibroblasts (OR 4.627, 95% CI 1.990–11.23, *p <* 0.001) to be significant risk factors for death because of lung cancer after surgery. However, multivariate analysis did not identify any independent risk factor for death from lung cancer after surgery (Additional file 2).

The DFS curves based on HSP47 expression status in cancer cells and the number of HSP47-positive fibroblasts in adenocarcinoma patients exhibited similar differences to those in all patients (Additional file 3). No significant differences were observed in either the DFS or OS curves based on HSP47 expression status in cancer cells and the number of HSP47-positive fibroblasts in patients with squamous cell carcinoma (data not shown).

## Discussion

The present study showed that patients with a higher number of HSP47-positive fibroblasts in the lung cancer stroma had a shorter DFS than those with few HSP47-positive fibroblasts. Logistic regression analysis identified the presence of a high number of HSP47-positive fibroblasts in the cancer stroma to be a significant risk factor for the recurrence of lung cancer after surgery, and multivariate analyses identified the presence of a higher number of HSP47-positive fibroblasts as the only independent risk factor for the recurrence of lung cancer after surgery. This is the first study demonstrating the association between HSP47 expression in lung cancer stroma and the recurrence of lung cancer after surgery.

Increased HSP47 expression in cancer cells promotes cancer progression in part by enhancing deposition of the ECM proteins [9], and several types of cancers are correlated with HSP47 expression in cancer cells [8–10]. The present study demonstrated that HSP47-positive fibroblasts in the stroma, but not HSP47-positive cancer cells, were associated with recurrence of lung cancer after surgery. Fibroblasts in solid tumors are known as carcinoma-associated fibroblasts (CAFs) [17, 18]. These arise from bone marrow-derived precursors and/or tissue-resident stromal cells through cancer cell-induced reprogramming and can be phenotypically identified using markers such as alpha smooth muscle actin (αSMA), which is a marker of differentiated and activated fibroblasts called myofibroblasts [19]. Based on paracrine and juxtracrine signals, these stromal cells modulate cancer progression both directly and indirectly [17, 20]. Furthermore, the production and deposition of excessive ECM, commonly known as fibrosis, can also foster tumor progression, even though the underlying mechanisms remain to be fully elucidated [21]. In non-small-cell lung carcinoma cells, pirfenidone, a pyridine compound with therapeutic potential for idiopathic pulmonary fibrosis, significantly inhibits fibrosis and decreases tumor growth when used in combination with carboplatin [22]. These results suggest that fibrosis plays a pivotal role in the development of lung cancer. Furthermore, the relationship between HSP47 expression and lung fibrosis has been reported previously [3–7, 23–25], and HSP47 expression was associated with αSMA-positive fibroblasts in animal models of, and humans with, pulmonary fibrosis [3, 5]. These results suggest that the HSP47-positive fibroblasts in the present study represent CAFs. Collagen is the major component of ECM and plays a critical role in the tumor microenvironment [17]. HSP47 is essential for correct folding and secretion of collagen. Xu et al. reported that HSP47-positive stromal cell levels in patients with obstructing colon cancers were higher than those in non-obstructing colon cancers [26]. Mori et al. reported that HSP47-positive spindle cells serve as independent biomarkers for DFS and OS of patients with colorectal cancer [11]. They also showed that tumor budding, which represents a highly invasive phenotype, was associated with HSP47-positive spindle cells [11, 26]. In addition, HSP47 is reported to be expressed in the tumor-associated stromal desmoplasia of patients with pancreatic carcinoma, but HSP47 expression is absent in the majority of nonneoplastic pancreas [27]. Hirai et al. examined the HSP47 expression intensity in gastric cancer tissues, and found that the intensity of fibroblast staining was stronger than that of cancer cells [28]. These reports and our present study suggest that HSP47-positive fibroblasts might induce cancer progression and metastasis as CAFs and are associated with recurrence of lung cancer after surgery.

HSP47 expression in lung cancer cells was not a risk factor for reoccurrence and survival in the present study. HSP47 is encoded by the *SERPINH1* gene, which is located on chromosome 11q13.5, one of the most frequently amplified regions in human cancer [12]. Altered HSP47 expression levels have been reported in several types of cancer, and HSP47 expression in colorectal cancer cells was upregulated in patients with lymph node metastasis and in those with high HSP47 expression showed longer DFS [11]. However, another study showed that there were no differences in the number of HSP47-positive cancer cells between obstructing and non-obstructing colon cancers [26]. In patients with breast cancer, HSP47 expression was activated during breast cancer development and progression, and HSP47 promotes cancer progression by increasing cell proliferation and invasion [9]. One report stated that HSP47 was a common antigen expressed in pancreatic non-ductal neoplasms [29]. In non-small lung cancer cells, especially in squamous cell carcinomas, HSP47 expression was higher than that in the normal bronchial epithelium [14], although adenocarcinoma was the most common type of tumor in the present study. These reports and our present results suggest that the expression and roles of HSP47 in cancer cells, especially in a clinical setting, might differ according to the type of cancers.

This study has limitations that should be considered when interpreting the results. The present study included all types of lung cancers such as adenocarcinoma and squamous cell carcinoma. Although similar results were shown in the adenocarcinoma group, the meaning of HSP47 expression in each type of lung cancer was not examined separately. In addition, the mechanism and role of HSP47 expression in lung cancer progression was not demonstrated by in vitro studies. Further studies are needed to clarify the mechanism and the role of HSP47 in lung cancer progression.

## Conclusions

The present study demonstrated that the presence of a high number of HSP47-positive fibroblasts in the cancer stroma was a risk factor for recurrence of lung cancer after surgery.

## Supplementary information

**Additional file 1. **Survival curves of patients with lung cancer according to HSP47 expression or the number of HSP47-positive fibroblasts in patients with lung cancer. a) No significant difference in overall survival (OS) curves of patients showing HSP47-positive or HSP47-negative cancer cells (*p* = 0.388, log-rank test). b) Patients with a high number of HSP47-positive fibroblasts had significantly shorter OS (*p* < 0.001, log-rank test, cut-off value = 123).

**Additional file 2.** Risk factors for death

**Additional file 3. **Survival curves of patients with adenocarcinoma according to HSP47 expression in lung cancer or the number of HSP47-positive fibroblasts in the stroma. No significant difference in overall survival (OS). (a) and disease-free survival (DFS) (c) curves of patients showing HSP47-positive or HSP47-negative cancer cells (*p* = 0.260, *p* = 0.423, log-rank test). b) No significant difference in OS curves of patients with a high number of HSP47-positive fibroblasts and a low number of HSP47-positive fibroblasts (*p* = 0.524, log-rank test, cut-off value = 61). d) Patients with a high number of HSP47-positive fibroblasts had significantly shorter DFS (*p* = 0.012, log-rank test, cut-off value = 71).

**Additional file 4.** Risk factors for recurrence of adenocarcinoma

## Data Availability

All data generated or analysed during this study are included in this published article and its supplementary information files.

## Abbreviations

HSP47: Heat shock protein 47
ECM: Extracellular matrix
CAFs: Carcinoma-associated fibroblasts
αSMA: Alpha smooth muscle actin
IQR: Interquartile range
ROC: Receiver operating characteristic
OS: Overall survival
DFS: Disease free survival
OR: Odds ratio
CI: Confidence interval
