# Supplementary material for: Presence of heat shock protein 47-positive fibroblasts in cancer stroma is associated with increased risk of postoperative recurrence in patients with lung cancer
Source: Respir Res. 2020 Sep 14;21:234. doi: 10.1186/s12931-020-01490-1 (PMC7488681; doi:10.1186/s12931-020-01490-1)
Supplement: Supplementary file 1 — Additional file 1. Survival curves of patients with lung cancer according to HSP47 expression or the number of HSP47-positive fibroblasts in patients with lung cancer. a) No significant difference in overall survival (OS) curves of patients showing HSP47-positive or HSP47-negative cancer cells (p = 0.388, log-rank test). b) Patients with a high number of HSP47-positive fibroblasts had significantly shorter OS (p < 0.001, log-rank test, cut-off value = 123). [file 12931_2020_1490_MOESM1_ESM.pptx]

## Slide 1
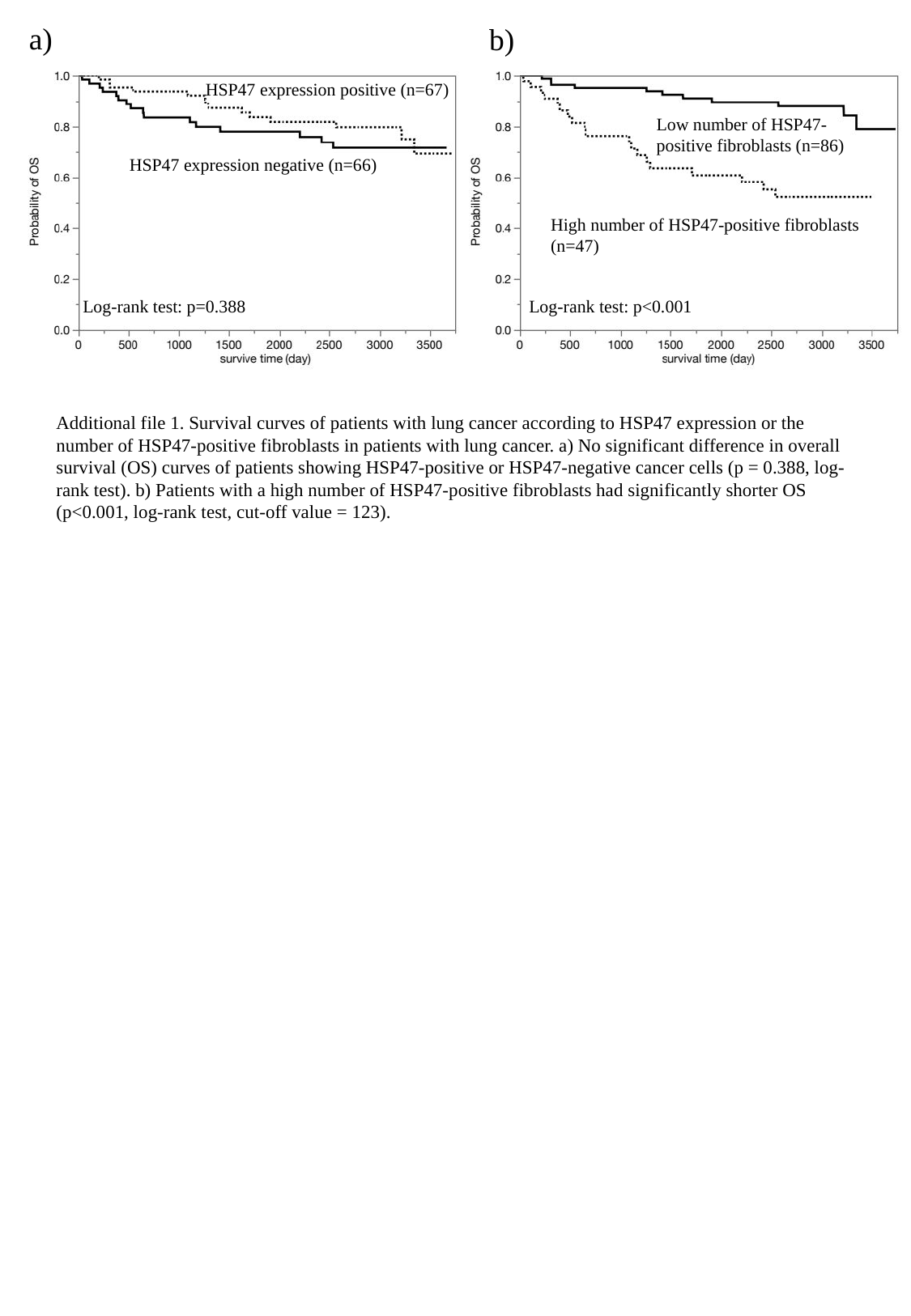

a)
b)
HSP47 expression positive (n=67)
Low number of HSP47-
positive fibroblasts (n=86)
High number of HSP47-positive fibroblasts (n=47)
Log-rank test: p<0.001
HSP47 expression negative (n=66)
Log-rank test: p=0.388
Additional file 1. Survival curves of patients with lung cancer according to HSP47 expression or the number of HSP47-positive fibroblasts in patients with lung cancer. a) No significant difference in overall survival (OS) curves of patients showing HSP47-positive or HSP47-negative cancer cells (p = 0.388, log-rank test). b) Patients with a high number of HSP47-positive fibroblasts had significantly shorter OS (p<0.001, log-rank test, cut-off value = 123).
