# Supplementary material for: Presence of heat shock protein 47-positive fibroblasts in cancer stroma is associated with increased risk of postoperative recurrence in patients with lung cancer
Source: Respir Res. 2020 Sep 14;21:234. doi: 10.1186/s12931-020-01490-1 (PMC7488681; doi:10.1186/s12931-020-01490-1)
Supplement: Supplementary file 3 — Additional file 3. Survival curves of patients with adenocarcinoma according to HSP47 expression in lung cancer or the number of HSP47-positive fibroblasts in the stroma. No significant difference in overall survival (OS). (a) and disease-free survival (DFS) (c) curves of patients showing HSP47-positive or HSP47-negative cancer cells (p = 0.260, p = 0.423, log-rank test). b) No significant difference in OS curves of patients with a high number of HSP47-positive fibroblasts and a low number of HSP47-positive fibroblasts (p = 0.524, log-rank test, cut-off value = 61). d) Patients with a high number of HSP47-positive fibroblasts had significantly shorter DFS (p = 0.012, log-rank test, cut-off value = 71). [file 12931_2020_1490_MOESM3_ESM.pptx]

## Slide 1
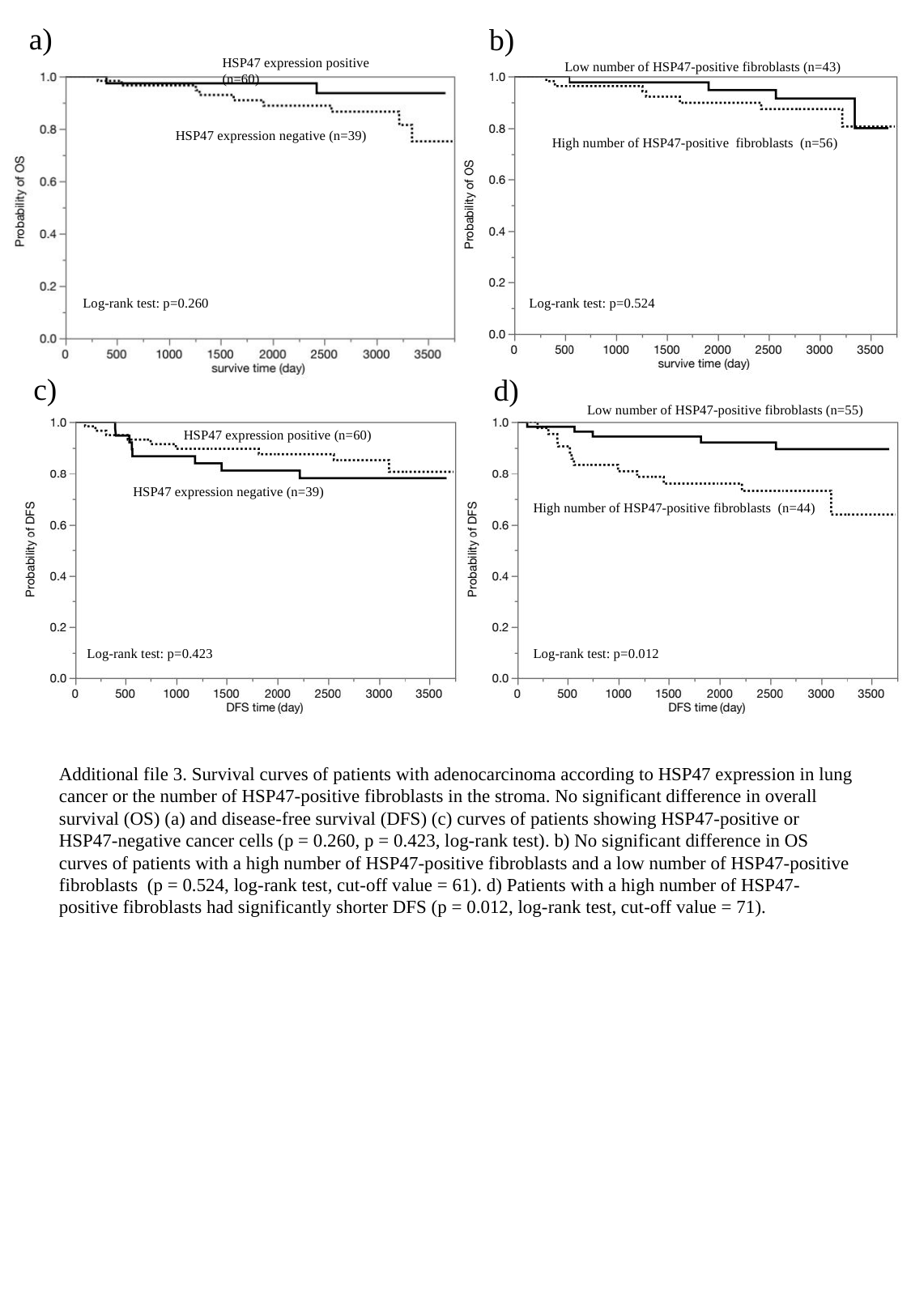

a)
b)
HSP47 expression positive (n=60)
Low number of HSP47-positive fibroblasts (n=43)
High number of HSP47-positive fibroblasts (n=56)
Log-rank test: p=0.524
HSP47 expression negative (n=39)
Log-rank test: p=0.260
c)
d)
Low number of HSP47-positive fibroblasts (n=55)
High number of HSP47-positive fibroblasts (n=44)
Log-rank test: p=0.012
HSP47 expression positive (n=60)
HSP47 expression negative (n=39)
Log-rank test: p=0.423
Additional file 3. Survival curves of patients with adenocarcinoma according to HSP47 expression in lung cancer or the number of HSP47-positive fibroblasts in the stroma. No significant difference in overall survival (OS) (a) and disease-free survival (DFS) (c) curves of patients showing HSP47-positive or HSP47-negative cancer cells (p = 0.260, p = 0.423, log-rank test). b) No significant difference in OS curves of patients with a high number of HSP47-positive fibroblasts and a low number of HSP47-positive fibroblasts (p = 0.524, log-rank test, cut-off value = 61). d) Patients with a high number of HSP47-positive fibroblasts had significantly shorter DFS (p = 0.012, log-rank test, cut-off value = 71).
